# Smoothness Metrics in Complex Movement Tasks

**DOI:** 10.3389/fneur.2018.00615

**Published:** 2018-09-12

**Authors:** Philipp Gulde, Joachim Hermsdörfer

**Affiliations:** Sports and Health Sciences, Technical University of Munich, Munich, Germany

**Keywords:** activity of daily living, smoothness, kinematics, number of peaks, spectral arc length, speed metric, jerk

## Abstract

Smoothness is a main characteristic of goal-directed human movements. The suitability of approaches quantifying movement smoothness is dependent on the analyzed signal's structure. Recently, activities of daily living (ADL) received strong interest in research on aging and neurorehabilitation. Such tasks have complex signal structures and kinematic parameters need to be adapted. In the present study we examined four different approaches to quantify movement smoothness in ADL. We tested the appropriateness of these approaches, namely the number of velocity peaks per meter (NoP), the spectral arc length (SAL), the speed metric (SM) and the log dimensionless jerk (LDJ), by comparing movement signals from eight healthy elderly (67.1a ± 7.1a) with eight healthy young (26.9a ± 2.1a) participants performing an activity of daily living (making a cup of tea). All approaches were able to identify group differences in smoothness (Cohen's d NoP = 2.53, SAL = 1.95, SM = 1.69, LDJ = 4.19), three revealed high to very high sensitivity (z-scores: NoP = 1.96 ± 0.55, SAL = 1.60 ± 0.64, SM = 3.41 ± 3.03, LDJ = 5.28 ± 1.52), three showed low within-group variance (NoP = 0.72, SAL = 0.60, SM = 0.11, LDJ = 0.71), two showed strong correlations between the first and the second half of the task execution (intra-trial R^2^s: NoP = 0.22 n.s., SAL = 0.33, SM = 0.36, LDJ = 0.91), and one was independent of other kinematic parameters (SM), while three showed strong models of multiple linear regression (R^2^s: NoP = 0.61, SAL = 0.48, LDJ = 0.70). Based on our results we make suggestion toward use examined smoothness measures. In total the log dimensionless jerk proved to be the most appropriate in ADL, as long as trial durations are controlled.

## Introduction

Despite the great importance of analyzing ecologically valid activities in clinical research and diagnostics, the quantification of activities of daily living (ADL) was typically limited to subjective scorings of videos (1, 2) or timed trials (3–5), although a parametric quantification of movement quality was not feasible. With technologies like advanced motion-tracking devices, it recently became more feasible to investigate human behavior in a natural, ecological valid setting. In the performance of such tasks, goals can be achieved in various ways and actions can't be precisely predicted (6). However, certain actions, like phases of inactivity, transporting, grasping, rotating, circling, or balancing, repeatedly appear in ADL. In kinematic analyses, smoothness is a main characteristic of goal-directed movements. It is suggested that the planning of movement dynamics are based on smoothness (7). Thus, deficits in planning are reflected in reduced movement smoothness. Consequently, the assessment of smoothness has become a central metric for the diagnosis and supervision of motor rehabilitation in neurological disease. Therefore research, especially in upper-limb rehabilitation in stroke survivors, shows interest in quantifying smoothness (8–13).

For different types of movement tasks, trajectories and corresponding velocity profiles differ in the signals' structures (14). Assessing complex tasks, like ADL, the signal can show peculiarities like phases of inactivity or different quantities of actions (6, 15). This can lead to misestimations, when using approaches that utilize data processing that is developed for less complex signals, which can be periodic (16, 17) [brushing teeth, sawing, or hammering (18)] or of short lengths (8) [reaching or grasping movements (8, 13)]. Common smoothness parameters can be of basically three different types: Velocity-based parameters, acceleration-based parameters and arc-length-based parameters. The present classification bases on the differentiation between velocity peak, different kinds of jerk and spectral analysis metrics (12, 17, 19), whereas spectral analysis metrics follow the assumptiom that less smooth movements are more complex in terms of their frequency composition when approximating the original signal with a Fourier series [for an easy introduction to spectral analysis see (20)]. Various velocity-based parameters were suggested: the number of velocity peaks (6, 21–23), the normalized average speed (12, 24), the relative level of activity (6, 21, 24, 25), or the peak-based composition of the velocity profile (12, 26). Acceleration-based parameters are examining the rate of change of movement acceleration and are usually forms of the jerk metric like the normalized jerk (27, 28), the log dimensionless jerk (8), the normalized squared jerk (14), or the normalized mean jerk (12). The arc-length-based parameters measure movement smoothness by defining the complexity of the signal by the arc length of its profile, that is the velocity profile (8) or the profile of its power spectrum (8, 17). So far, the were several attempts to compare and classify smoothness metrics, including investigations in a stroke samples (8, 12), a sample of patients with cerebral palsy (13), and of a general kind (17). However this research focused on mainly reaching movements and did not examine the behavior of smoothness metrics when confronted with the complex signals of ADL performance in healthy elderly. It has been shown that even in healthy elderly movement smoothness is decreased (29–31), however, not a drastically as in neurological patients.

In this study we assessed the validity of different smoothness parameters by comparing the hand movements of young and elderly participants in an ADL. We investigated to what degree the used parameters were sensitive, variable, and independent of general movement characteristics like velocities, trial durations, path lengths, or the activity level. Further, we examined if the parameters are quantifying a general ability to produce smooth movements. ADL offer a way to examine behavior in the context of disease and aging in an ecological valid way and should therefore be considered in research and clinical assessment (6, 10, 11, 21, 32). This study tries to explore the behavior of different parameters in an empirical way in order to give suggestions and an outlook on future developments.

## Methods

We compared the movements of the dominant hand of 8 healthy young adults (26.9a ± 2.1a) with the movements of the dominant hand of 8 healthy elderly participants (67.1a ± 7.1a). The ADL task was to unimanually prepare a cup of tea with milk and sugar (21). All participants were right handed and each participant executed the task once. Ethical approval was obtained by the local ethics committee of the Medical Faculty of the Technical University of Munich. All subjects provided written informed consent.

The experimental set-up was similar to the one in Gulde et al. (21). Participants stood in front of a table with the following items placed on its surface in a semi-circular order from left to right: a container with room temperatured water, a milk carafe, a saucer for used tea-bags, an open container with tea-bags, an open container with sugar cubes, an open container with coffee powder (as a distractor item), and an empty kettle. Additional and in front, there were a mug and spoon located on the table. Participants were asked to execute the task in a natural way without emphasis on speed.

The positional data of the dorsum of the hand was obtained by a Qualisys motion capturing system (Qualisys Inc. Gothenburg, Sweden) incorporating 5 Oqus cameras sampling with a frequency of 120 Hz. There were no gaps in the recordings. All post-processing was computed with MatLab (MATLAB R2017a, MathWorks, MA). After differentiation the data were smoothed using a 0.1s local regression filter (“loess”) (33). This short smoothing window (12 frames) was chosen in order to preserve as much information as possible, to not corrupt the outcomes of spectral analysis, and in order to not fully eliminate noise, which can have a specific impact on jerk metrics (17). The time to boil the water, being dependent on water temperature and filling level, was not excluded from the signal (typically no hand movements occur in this waiting period). This was done in order to provoke meaningless variance in the signal and activity index (relative amount of time with hand movements).

The used smoothness parameters were the spectral arc length (8), the (negative–see description) number of velocity peaks per meter (21), the speed metric (12) and the log dimensionless jerk (8). The spectral arc length is calculated from the arc length of the power spectrum of a Fourier transformation of the velocity signal (8).


$$\begin{array}{l} spectral\;arc\;length≜-{\int^{\text{​}}}_{\omega c}^{0}\sqrt{{\left(\frac{1}{\omega c}\right)}^{2}+{\left(\frac{d\hat{V}(\omega )}{d\omega }\right)}^{2}}\;d\omega ,\\ \;\hat{V}(\omega )≜\frac{V(\omega )}{V(0)} \end{array}$$

Calculation of the spectral arc length based on a velocity profile v, with [0, ω*c*] being the frequency band and V(ω) the Fourier magnitude spectrum (8).

For the number of velocity peaks per meter all peaks of the velocity profile, which exceed a prominence of 0.05 m/s, are counted and divided by the traveled path length. The resulting number is inverted, so that higher values indicate smoother movements (21).


number of velocity peaks per meter≜−peaks/∫v

Calculation of the number of velocity peaks per meter based on a velocity profile v and peaks being maxima with a prominence exceeding 0.05 m/s (21).

The speed metric is obtained by dividing the average velocity by the maximum velocity (12).


speedmetric≜Θv/max(v)

Calculation of the speed metric based on a velocity profile v (12).

The log dimensionless jerk results from the logarithm naturalis of the sum of the squared acceleration multiplied with the trial duration to the power of three and divided by the squared peak velocity (8).


$$\begin{array}{l} \log\text{dim}.\text{ jerk}≜-\ln\left(\frac{{\left(t_{2}-t_{1}\right)}^{3}}{v_{peak}^{2}}\int_{t_{1}}^{t_{2}}|\frac{d^{2}v}{dt^{2}}{|}^{2}dt\right) \end{array}$$

Calculation of the log dimensionless jerk based on a velocity profile *v* with the time window *t*_1_ to *t*_2_ (8).

Note that all parameters but the speed metric output negative values, and for every parameter values closer to zero represent smoother movements. These four parameters were considered as prototypical agents for the different classes of smoothness measures listed in the introduction. The speed metric was added, since its computation strongly differs from the peaks metric and therefore its behavior could not have been derived.

The smoothness parameters were compared between groups using *t*-tests for independent samples with α = 0.05. Effect-sizes were calculated with Cohen's d (34). Sensitivity and within-group variability were analyzed on the basis of z-scores (reference: distribution of elderly subjects). Sensitivity was expressed as the mean z-score and within-group variability was calculated by *1-std(z-scores)/mean(z-scores)* (with = 1 being perfectly stable and < 0 being perfectly unstable). The z-standardization was performed in order to be able to compare the sensitivity between the different parameters. Multiple linear regression models were applied to check for independence from the following kinematic parameters: trial duration (21), path length (21), mean peak velocity (average of velocity peaks with a minimum prominence of 0.05 m/s) (21), and relative activity (the relative amount of the trial duration in which the hands rest, defined by a velocity below 0.05 m/s) (21). The critical variance inflation factor (VIF) was set to 5. To avoid a moderation of age, the models of multiple linear regression were based on z-scores (within-group). Further, the trials were split into two halves (on the basis of trial duration) and the first half was compared to the second half of the trial (intra-individually) by correlational analyses. This can be considered as an alternative form of test-retest reliability, since by splitting the task into halves we can examine the within-subject behavior over two different tasks—which can be considered an increased generalizability of the outcomes. Of course, this lacks the comparison between two different time periods of the same task, but this would be prone to learning effects (and moderations by age) and therefore changes in movement smoothness in such a complex task and the reliability of a classic test-retest approach would therefore be questionable. Additionally, the four parameters were correlated with each other in order to get an estimate, if they are generally measuring the same phenomenon. The effect-sizes of the regressions were defined according to Cohen (34) as *r* > 0.1 being weak, *r* > 0.3 being moderate and *r* > 0.5 being strong. The small sample size can impact the outcomes of the statistical tests, especially of the models of multiple linear regression. The impact of variables can be therefore misestimated. The statistical power for all tests was determined *post-hoc* with a critical power of 0.80 using G^*^Power (G^*^Power 3.192, 2014, HHU Düsseldorf, Germany).

## Results

The comparison of the groups revealed significant differences for all four parameters (Table 1) reaching from Cohen's ds of 1.69–4.19. In all cases the young participants' movements were classified as being smoother. The sensitivity was measured by the z-scores of the young participants referenced to the elderly participants. Table 2 contains the resulting magnitudes of sensitivity and within-group variability for the different parameters. Sensitivity was calculated 1.60–5.28 and within-group variability 0.11–0.72. The results of the comparison of the first half with the second half of each trial are covered by Table 3, with R^2^s ranging from 0.22 to 0.91. Note, that only the speed metric and the log dimensionless jerk reached significance and delivered power estimates of at least 0.80. The models of multiple linear regression showed significant models for number of velocity peaks per meter, spectral arc length, and log dimensionless jerk. The models for speed metric were all non-significant (Table 4). The correlations between the parameters were, except log dimensionless jerk & speed metric, all significant and strong (Table 5).

**Table 1:** The means, standard deviations, effect-sizes, *p*-values, and power estimates for the young and the elderly sample for the four smoothness parameters.

	**Young**	**Elderly**	***p*-value**	**Cohen's d**	**Power**
Number of peaks per meter	−5.43 ± 0.67	−7.82 ± 1.22	< 0.01	2.53	1.00
Spectral arc length	−12.55 ± 2.16	−17.97 ± 3.39	< 0.01	1.95	0.95
Speed metric	0.12 ± 0.04	0.08 ± 0.01	0.02	1.69	0.88
Log dimensionless jerk	−12.03 ± 0.70	−14.47 ± 0.46	0.01	4.19	1.00

**Table 2:** The means and standard deviations for the sensitivity measure and the within-group variability index for the four smoothness parameters.

	**Sensitivity**	**Within-group variability**
Number of peaks per meter	1.96 ± 0.55	0.72
Spectral arc length	1.60 ± 0.64	0.60
Speed metric	3.41 ± 3.03	0.11
Log dimensionless jerk	5.28 ± 1.52	0.71

**Table 3:** The *R*^2^-values of the intra-trial correlation, its *p*-values and power estimates for the four smoothness parameters.

	**Intra-trial correlation**	***p*-value**	**Power**
Number of peaks per meter	*R*^2^ = 0.22	0.07	
Spectral arc length	*R*^2^ = 0.33	0.02	0.75
Speed metric	*R*^2^ = 0.36	0.01	0.80
Log dimensionless jerk	*R*^2^ = 0.91	< 0.01	1.00

**Table 4:** The corrected *R*^2^-values of models of multiple linear regression, their p-values, power estimates, and the models' factors for three of the four smoothness parameters.

	**Corrected *R*^*2*^**	***p*-value**	**power**	**Factors**
Number of peaks per meter	*R*^*2*^ = 0.61	< 0.01	0.98	Path length (ß = −0.36, *p* = 0.05) Mean peak velocity (ß = 0.66, *p* < 0.01)
Spectral arc length	*R*^*2*^ = 0.48	< 0.01	0.87	Trial duration (ß = −0.86, *p* < 0.01) Mean peak velocity (ß = 0.60, *p* = 0.02)
Log dimensionless jerk	*R*^*2*^ = 0.70	< 0.01	1.00	Trial duration (ß = −0.85, *p* < 0.01)

*For the speed metric, none of the models was significant*.

**Table 5:** Correlations between the four different smoothness parameters.

	**Number of peaks per meter**	**Spectral arc length**	**Speed metric**
Log dimensionless jerk	*r* = 0.79 (*p* < 0.01, power = 1.00)	*r* = 0.77 (*p* < 0.01, power = 0.99)	*r* = 0.50 (*p* = 0.051)
Speed metric	*r* = 0.57 (p = 0.02, power = 0.73)	*r* = 0.71 (*p* < 0.01, power = 0.96)	
Spectral arc length	*r* = 0.58 (p = 0.02, power = 0.75)		

## Discussion

In the present study, we analyzed movement smoothness that is known as a highly characteristic aspect of task performance. Since measures of smoothness were typically established for simple continuous or discrete movements, we here analyzed the suitability of various measures for the evaluation of the complex activity of daily living of tea making.

The comparison of the four smoothness parameters revealed that all of the methods were able to detect the differences in smoothness between young and elderly participants in the ADL of tea making. With mean z-scores between 1.60 and 5.28 all four parameters proved to be highly sensitive. Three of the parameters showed a within-group variance index above or equal 0.6, meaning that within group variability was low in the number of velocity peaks per meter, the spectral arc length, and the log dimensionless jerk, while it was very high in the speed metric. The intra-trial comparisons (first half vs. second half) further revealed that three of the parameters were significantly correlated between the two halves with the strength of the correlations being strong. Note, that in one case the statistical power was lower than 0.80 (spectral arc length). High correlations between the two halves support a generalization beyond this specific ADL. By splitting, two different tasks were artificially created and in case of high intra-trial correlations, the metric shows the capability to estimate the participant's general and not task restricted movement smoothness. Lastly, the models of multiple linear regression revealed an impact of kinematic parameters on three of the parameters. All of the models were strong (*r* > 0.5), although the sample size was small with 16 participants. The small sample size could have led to missing a possible dependence of the speed metric on kinematic parameters. Each of the four parameters was strongly connected with at least one other smoothness parameter (Table 5), leading to the assumption that the used parameters are basically measuring the same phenomenon.

Table 6 provides an overview of the outcomes of the parameter analysis.

**Table 6:** Color-coded overview of the interpreted outcomes of the parameter analysis.

	**Group differences detected**	**Sensitivity**	**Within-group variance**	**Intra-trial correlation**	**Dependence on kinematic parameters**
Number of velocity peaks per meter	Yes	High	Low	No	Strong (–) path length (+) mean peak velocity
Spectral arc length	Yes	High	Low	Strong (power < 0.8)	Strong (–) trial duration (+) mean peak velocity
Speed metric	Yes	Very high	Very high	Strong	None
Log dimensionless jerk	Yes	Very high	Low	Strong	Strong (–) trial duration

*Green refers to good characteristics, yellow to acceptable characteristics and red to weaknesses*.

*Sensitivity was defined as 0.5 ≤ × < 1.0 being moderate, 1.0 ≤ × < 2.0 being high and x ≥ 2.0 being very high. Within-group variance was defined as x ≤ 0 being perfectly unstable, 0 < × < 0.2 being very high, 0.2 ≤ × < 0.4 being high, 0.4 ≤ × < 0.6 being moderate, 0.6 ≤ × < 0.8 being low and 0.8 ≤ × being very low*.

Of the four parameters, none proved to be fully suited for a general quantification of smoothness in the tested ADL, although log dimensionless jerk did reveal good characteristics except its very strong association with trial duration. The number of velocity peaks per meter was able to detect the group differences, showed high sensitivity, low within-group variance, but the correlation between the first and the second half of the trial was non-significant (although a trend was observed, *p* = 0.07) and the model of multiple linear regression revealed a strong dependence on the traveled path length and mean peak velocity. The impact of mean peak velocity on smoothness can be explained by the fact that smooth movements promote faster movements and the change in the signal-to-noise ratio (17). The dependence on path length is surprisingly not an artifact of the calculation method of the parameter. The impact was negative, which means that the smoothness decreases with longer trajectories. Smaller movements, like repetitive approaches to the mug or hesitating and retrieving the hand could explain this: They produce peaks with small movements and at the same time add (unnecessary) path length. This would make the parameter better suited for the comparison of groups with similar path lengths and a comparable number of actions, this is for instance given in stroke patients and age-matched controls, who revealed comparable path lengths and action steps in the ADL of tea making (6). The spectral arc length was also able to detect the group differences, proved to be highly sensitive, revealed a low within-group variance, but showed a correlation in the intra-trial comparison with a power lower than 0.80, and revealed a strong dependence on kinematic parameters. Trial duration negatively impacted the model, resulting in less smooth movements in longer trials. The basis could be that trial duration in both groups was strongly associated with the general motor capability—this has been at least reported in stroke patients (10). The model was also impacted by the mean peak velocity, same as in the model for the number of velocity peaks per meter (with comparable ßs of 0.60 and 0.66). This would make the parameter well suited for comparisons with equal trial durations and movement speeds (also see power of intra-trial correlation). Unimanual ADL performance with the dominant and the non-dominant hand would be a possibility, where young as well as elderly revealed no difference in mean peak velocity (21). The speed metric was able to detect to group differences, proved highly sensitive and had a strong correlation in the intra-trial comparison, but revealed a very high within-group variance. It further proved to be independent of other commonly used kinematic parameters, although this could be due to the small sample size. It is advisable to use this parameter with caution, due to its high within-group variance. It appeared to be not suited for an analysis of the ADL. The log dimensionless jerk was able to detect the group differences, proved highly sensitive and was strongly correlated in the intra-trial comparison. It further revealed a low within-group variance, but a strong dependence on the trial duration. The impact of trial duration was negative, same as for the spectral arc length. The log dimensionless jerk appeared to be well suited for the analysis of ADL, as long as the variability in the trial duration is controlled for. Although trial duration and movement capacity can be associated in patient groups (10), there is evidence that trial durations in ADL are based on other factors like movement strategy or cognitive factors in healthy adults (21), therefore controlling for trial duration in spectral arc length as well a log dimensionless jerk appear substantial.

## Conclusions

The analysis of the four smoothness parameters revealed that there is still the necessity for a novel, well-suited parameter for the analysis of movement smoothness in ADL. Still, three of the four parameters proved to deliver good estimates, when controlling for certain aspects of an experiment. Since the sample size was relatively small, our findings have to be interpreted with care, although the statistical power was mostly high. In addition, we tested only one ADL task and the question is how much our findings can be extended to ADL in general. However, certain patterns and combinations of actions, for instance phases of inactivity, grasping, or transporting, repeatedly appear in ADL. This is particularly true in an ADL that demands manual interactions with serval objects like the one analyzed here. We therefore believe that our findings can be generalized to a broad class of ADL and draw the following recommendations for the use of smoothness parameters in ADL: The number of velocity peaks per meter needs comparisons with equal lengths of the trajectories and comparable quantities of performed actions in a task. The spectral arc length needs tasks with comparable trial durations and movement speeds. The log dimensionless jerk, having a very strong intra-trial correlation, very high sensitivity, low within-group variability, but a very strong dependence on trial duration, should deliver good estimates on a wide range of ADL tasks, as long as the trial durations are controlled for. Given these prerequisites, these three parameters (number of velocity peaks per meter, spectral arc length & log dimensionless jerk) can deliver appropriate estimates of movement smoothness in complex motor tasks like ADL. Future research should examine the different sub-types of those measures and different ADL tasks to see if the behavior of the parameter class and the ADL (tea making) are generalizable.

The search for a universal smoothness parameter for ADL should have a high priority in neurorehabilitational research in order to assess motor capacity and supervise the rehabilitation process of patients suffering from neurological diseases like stroke, Parkinson's disease, or cerebral palsy. So far, comparisons of movement smoothness with control groups or in patient groups with high (kinematic) variabiliy and during the supervision of the rehabilitation process with changes in trial durations, mean peak velocities, or path lengths, are limited. A promising approach could be using wavelet transformation in order to estimate the complexity of the signal (analog to the spectral arc length metric).

## Limitations

There are clear limitations in this study. Although examining an ADL, ecological validity was limited by the unimanual execution of the task. However, previous research has shown that the transfer from bimanual to unimanual performance has no interaction with age (21), and the unimanual execution controlled for variability in hand use in bimanual conditions. Considering the relatively small sample size, appropriate statistical power was still given. Further, the examination of a non-standardized, complex task with parameters that are fitted to quantify movement smoothness in discrete, single movement or cyclic tasks without further data processing like segmentation is a questionable approach considering validity and reliability. However, the results of this study strongly suggest that it is indeed possible to quantify movement smoothness in ADL by the existing parameter classes, although adaptations are still necessary. Another limitation is the small sample size. Therefore some associations could have been underestimated (e.g., the dependence of the speed metric on other kinematic parameters). Still, the analyses were able to reveal certain associations and the power estimates were acceptably high. Last, an assessment of test-retest reliability was only partially possible, since in such complex task fast adaptations through learning can be expected, as well as moderations of learning rates by age.

## Author contributions

PG and JH designed the study. PG performed the lab testing and the kinematic and statistical analyses. All authors contributed to the coordination of the study and the final draft of the manuscript.

### Conflict of interest statement

The authors declare that the research was conducted in the absence of any commercial or financial relationships that could be construed as a potential conflict of interest.

## References

[1] Buxbaum L. Ideational apraxia and naturalistic action. Cogn Neuropsychol. (1998) 15:617–43. 10.1080/026432998381032 22448839

[2] Giovannetti T Libon DJ Buxbaum LJ Schwartz MF. Naturalistic action impairments in dementia. Neuropsychologia (2002) 40:1220–32. 10.1016/S0028-3932(01)00229-9 11931925

[3] Bickerton W Riddoch MJ Samson D Balani AB Mistry B Humphreys GW. Systematic assessment of apraxia and functional pedictions from the Birmingham Cognitive Screen. J Neurol Neurosurg Psychiatry Pract Neurol. (2012) 85:512–52. 10.1136/jnnp-2011-300968 22383734

[4] Ehrensperger M Berres M Taylor KI Monsch AU. Early detection of Alzheimer's disease with a total score of the German CERAD. J Int Neuropsychol Soc. (2010) 16:910–20. 10.1017/S1355617710000822 20682088

[5] Carr D Barco PP Wallendorf MJ Snellgrove CA Ott BR. Predicting road test performance in drivers with dementia. J Am Geriatric Soc. (2011) 59:2112–7. 10.1111/j.1532-5415.2011.03657.x 22092029 PMC3228409

[6] Gulde P Hughes C Hermsdörfer J. Effects of stroke on ipsilesional end-effector kinematics in a multi-step activity of daily living. Front Hum Neurosci. (2017) 11:42. 10.3389/fnhum.2017.00042 28223927 PMC5293874

[7] Cruse H. On the cost functions for the control of the human arm movement. Biol Cybernet. (1990) 62:519–28. 10.1007/BF00205114 2357475

[8] Balasubramanian S Melendez-Calderon A Burdet E. A robust and sensitive metric for quantifying movement smoothness. IEEE Trans Biomed Eng. (2012) 59:2126–36. 10.1109/TBME.2011.2179545 22180502

[9] Alt Murphy M Willén C Sunnerhagen K. Kinematic variables quantifying upper-extremity performance after stroke during reaching and drinking from a glass. Neurorehabil Neural Rep. (2011) 25:71–80. 10.1177/1545968310370748 20829411

[10] Alt Murphy M Willén C Sunnerhagen K. Movement kinematics during a drinking task are associated with the activity capacity level after stroke. Neurorehabil Neural Rep. (2012) 26:1106–15. 10.1177/1545968312448234 22647879

[11] Alt Murphy M Willén C Sunnerhagen K. Responsiveness of upper extremity kinematic measures and clinical improvement during the first three months after stroke. Neurorehabil Neural Rep. (2013) 27:844–53. 10.1177/1545968313491008 23764883

[12] Rohrer B Fasoli S Krebs HI Hughes R Volpe B Frontera WR. Movement smoothness changes during stroke recovery. J Neurosci. (2002) 22:8297–304. 10.1523/JNEUROSCI.22-18-08297.2002 12223584 PMC6758113

[13] Rincón Montes V Quijano Y Chong Quero JE Villanueva Ayala D Pérez Moreno JC. Comparison of 4 different smoothness metrics for the quantitative assessment of movement's quality in the upper limb of subjects with cerebral palsy. In: Pan Ameican Health Care Exchanges (PAHCE). Brasilia: IEEE (2014).

[14] Hogan N Sternad D. Sensitivity of smoothness measures to movement duration, amplitude and arrests. J Motor Behav. (2009) 41:529–34. 10.3200/35-09-004-RC 19892658 PMC3470860

[15] Wood R. Task complexity: definition of the construct. Organ Behav Hum Decis Processes (1986) 37:60–82. 10.1016/0749-5978(86)90044-0

[16] Deeming T. Fourier analysis with unequally-spaced data. Astrophys Space Sci. (1975) 36:137–58. 10.1007/BF00681947

[17] Balasubramanian S Melendez-Calderon A Roby-Brami A Burdet E. On the analysis of movement smoothness. J Neuroeng Rehabil. (2015) 12:112. 10.1186/s12984-015-0090-9 26651329 PMC4674971

[18] Bieńkiewicz M Gulde P Goldenberg G Hermsdörfer J. Harmonicity of the movement as a measure of apraxic behaviour in stroke survivors, BIOSIGNALS 2014 - Proceedings of the International Conference on Bio-inspired Systems and Signal Processing. Angers: SciTePress (2014). p. 295–300. 10.5220/000491380295030

[19] de los Reyes-Guzmán A Dimbwadyo-Terrer I Trincado-Alonso F Monasterio-Huelin F Torricelli D Gil-Agudo A. Quantitative assessment based on kinematic measures of functional impairments during upper extremity movements: a review. Clin Biomech. (2014) 29:7119–727. 10.1016/j.clinbiomech.2014.06.013 25017296

[20] Stewart I. Seventeen Equations that Changed the World. London: Profile Books (2012).

[21] Gulde P Hermsdörfer J. Both hands at work: the effect of aging on upper-limb kinematics in a multi-step activity of daily living. Exp Brain Res. (2017) 235:1337–48. 10.1007/s00221-017-4897-4 28210758

[22] Hermsdörfer J Hentze S Goldenberg G. Spatial and kinematic features of apraxic movement depend on the mode of execution. Neuropsychologia (2006) 44:1642–52. 10.1016/j.neuropsychologia.2006.03.023 16678222

[23] Metrot J Mottet D Hauret I van Dokkum L Bonnin-Koang HY Torre K. Changes in bimanual coordination during the first 6 weeks after moderate hemiparetic stroke. Neurorehabil Neural Rep. (2013) 27:251–9. 10.1177/1545968312461072 23135767

[24] Adams R Lichter MD Krepkovich ET Ellington A White M Diamond PT. Assessing upper extremity motor function in practice of virtual acitivities of daily living. IEEE Trans Neural Syst Rehabil Eng. (2015) 23:287–96. 10.1109/TNSRE.2014.2360149 25265612 PMC4532436

[25] Beppu H Suda M Tanaka R. Analysis of cerebellar motor disorders by visually-guided elbow tracking movement. Brain (1984) 107:787–809. 10.1093/brain/107.3.787 6478179

[26] Baur B Fürholzer W Jasper I Marquardt C Hermsdörfer J. Effects of modified pen grip and handwriting training on writer's cramp. Arch Phys Med Rehabil. (2009) 90:867–75. 10.1016/j.apmr.2008.10.015 19406309

[27] Kim N Wininger M Craelius W. Training grip control with a Fitts' paradigm: a pilot study in chronic stroke. J Hand Ther. (2010) 23:63–71. 10.1016/j.jht.2009.10.004 20142007

[28] Romero D Van Gemmert AWA Adler CH Bekkering H Stelmach GE. Altered aiming movements in Parkinson's disease patients and elderly adults as a function of delays in movement onset. Exp Brain Res. (2003) 151:249–61. 10.1007/s00221-003-1452-2 12783145

[29] Contreras-Vidal J Teulings H Stelmach G. Elderly subjects are impaired in spatial coordination in fine motor control. Acta Psychol. (1998) 100:25–35. 9844554 10.1016/s0001-6918(98)00023-7

[30] Seidler-Dobrin R He J Stelmach G. Coactivation to reduce variability in the elderly. Motor Control (1998) 2:314–30. 10.1123/mcj.2.4.314 9758884

[31] Poston B Van Gemmert AW Barduson B Stelmach GE. Movement structure in young and elderly adults during goal-directed movements of the left and right arm. Brain Cogn. (2009) 69:30–38. 10.1016/j.bandc.2008.05.002 18556103 PMC2663567

[32] Obhi S. Bimanual coordination: an unbalanced field of research. Motor Control (2004) 8:111–20. 10.1123/mcj.8.2.111 15118197

[33] Gulde P Hermsdörfer J. A comparison of smoothing a filtering approaches using simulated kinematic data of human movements. In: Proceedings of the 11th International Symposium on Computer Science in Sport (IACSS 2017), Advances in Intelligent Systems and Computing. Vol. 663. Cham: Springer (2018).

[34] Cohen J. Statistical Power Analysis for the Behavioral Sciences. Hillsdale, NJ: Erlbaum. (1988).

