# Corrigendum: Smoothness metrics in complex movement tasks

**DOI:** 10.3389/fneur.2023.1279682

**Published:** 2023-09-14

**Authors:** Philipp Gulde, Joachim Hermsdörfer

**Affiliations:** Sports and Health Sciences, Technical University of Munich, Munich, Germany

**Keywords:** activity of daily living, smoothness, kinematics, number of peaks, spectral arc length, speed metric, jerk

In the published article, there was an error. In the formula for the log dimensionless jerk, a ^3^ (to the power of three) was missing.

A correction has been made to the Methods section, fourth formula. This formula previously stated:


$$\begin{array}{l} \log\text{dim}.\text{ jerk}≜-ln\left(\frac{\left(t^{2}-t^{1}\right)}{v_{peak}^{2}}\int_{t_{1}}^{t_{2}}|\frac{d^{2}v}{dt^{2}}{|}^{2}dt\right) \end{array}$$

The corrected formula appears below:


$$\begin{array}{l} \log\text{dim}.\text{ jerk}≜-\ln\left(\frac{{\left(t_{2}-t_{1}\right)}^{3}}{v_{peak}^{2}}\int_{t_{1}}^{t_{2}}|\frac{d^{2}v}{dt^{2}}{|}^{2}dt\right) \end{array}$$

The authors apologize for this error and state that this does not change the scientific conclusions of the article in any way. The original article has been updated.

## Publisher's note

All claims expressed in this article are solely those of the authors and do not necessarily represent those of their affiliated organizations, or those of the publisher, the editors and the reviewers. Any product that may be evaluated in this article, or claim that may be made by its manufacturer, is not guaranteed or endorsed by the publisher.

